# A Rare Presentation of Sick Sinus Syndrome: Generalized Seizures

**DOI:** 10.7759/cureus.23631

**Published:** 2022-03-29

**Authors:** Sherif Elkattawy, Islam Younes, Ramez Alyacoub, Hardik Fichadiya, Preanka Dhanoa, Juliet Kotys, Omar Elkattawy, William Edward

**Affiliations:** 1 Internal Medicine, Trinitas Regional Medical Center, Elizabeth, USA; 2 Internal Medicine, St. George's University, Grenada, GRD; 3 Internal Medicine, Rutger New Jersey Medical Center, Newark , USA; 4 Cardiology, Trinitas Regional Medical Center, Elizabeth, USA

**Keywords:** tachycardia bradycardia syndrome, cardiogenic syncope, ictal bradycardia, sick sinus syndrome, seizures

## Abstract

Cardiac and neurological disorders are the main broad etiologies for loss of consciousness. Ictal bradycardia syndrome refers to epileptic discharges that profoundly disrupt normal cardiac rhythm, resulting in cardiogenic syncope during the ictal event. Convulsive syncope is a well-described phenomenon in both adults and children in which abrupt cerebral hypoperfusion leads to brief extensor stiffening and non-sustained myoclonus. Sick sinus syndrome or tachycardia bradycardia syndrome is a common cause of arrhythmias in the elderly secondary to sinus node dysfunction.

We present a case of a 91-year-old male who presented with generalized seizure with associated bradyarrhythmias with telemetry showing sinus rhythm, followed by severe bradycardia, followed by Ventricular tachycardia, followed by an episode of asystole, which likely precipitated seizures as a result of cerebral hypoperfusion. The patient had a permanent dual-chamber pacemaker. He was discharged on antiepileptics as his EEG was abnormal, which might indicate an underlying predisposition.

## Introduction

Episodes of transient loss of consciousness are commonly due to syncope or epileptic seizures. It is sometimes challenging to differentiate between entities as one can lead to another. Ictal bradycardia refers to bradycardia that occurs during seizure attacks. This has been explained by changes in the autonomic nervous system functions during seizure episodes [1]. On the other side, bradycardia can lead to seizures associated with prolonged brain hypoperfusion. Cardiac telemetry monitoring and electric brain monitoring by EEG are often required to be done together to catch the culprit pathology [2].

## Case presentation

This is a 91-year-old male with a history of coronary artery disease (status post multiple stents), mechanical aortic valve, and chronic thrombocytopenia presented to the emergency department after a presumed generalized seizure episode. Upon evaluation, the patient was seen after a dose of lorazepam and was severely lethargic. The patient could not provide a history, so it was obtained from the patient's daughter. She stated that her father complained of dizziness and was found to be confused the night before admission. The patient was taken to the emergency room and sent home the same evening with symptomatic treatment for nausea and emesis. The following morning the patient's mental status deteriorated; he developed difficulty with speech and eventually had a generalized seizure episode lasting one minute. During this time, the patient lost consciousness, had right tongue deviation, and was foamed from the mouth. There was no tongue biting or fecal or urinary incontinence reported. His mental status postictal was severely altered, so he arrived at the emergency department by EMS. 

In the ED, he had another episode of generalized seizure, for which he was given one dose of lorazepam. The patient was afebrile, and his vitals stable. His initial labs were significant for elevated lactate levels of 9.88 and a BNP of 614. Serum electrolytes were within normal limits. On telemetry in the ED, he had an episode of junctional bradycardia followed by asystole and eventually a non-sustained episode of ventricular tachycardia for 12 beats, which converted to normal sinus rhythm. 

**Figure 1:**
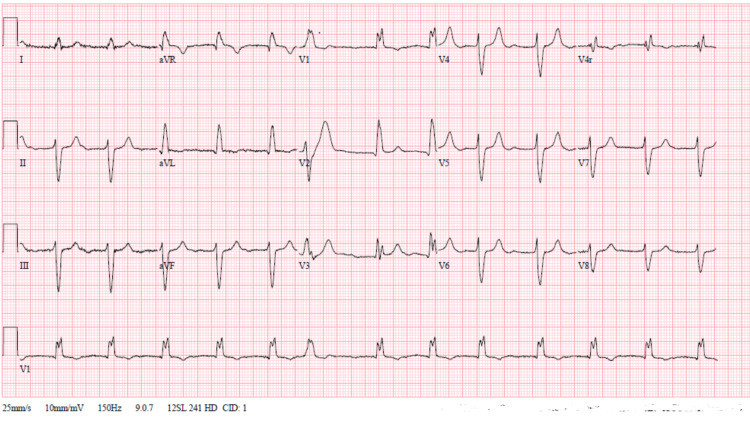
EKG showing Left anterior fascicular block, RBBB, and 1st degree AV block. RBBB- Right bundle branch block; AV- Atrioventricular

The patient's 12 lead ECG showed a right bundle branch block with left anterior hemiblock and prolonged PR interval significant for 1st degree atrioventricular block (figure 1).

The patient's head CT displayed multiple bilateral old basal ganglia lacunar infarcts with white matter changes, but there were no acute infarcts, intracranial hemorrhage, or masses noted. His EEG shows multiple spikes discharges consistent with an increased tendency for seizures. 

The patient was placed on the appropriate seizure and aspiration precautions and given one dose of Vancomycin and Piperacillin-Tazobactum in the ED. The patient's home medication, Propanolol 10mg, was held due to underlying bradyarrhythmia, and he was placed on Levetiracetam 500mg bid. The patient underwent temporary pacemaker placement and had a permanent pacemaker placed two days after admission. This patient could not have an MRI during his admission due to the pacemaker's recent placement and was scheduled to receive outpatient imaging. He had an MRI 6 months prior to admission that showed cerebral atrophy and chronic small vessel disease.

## Discussion

Sinoatrial (SA) node dysfunction is common due to age-related changes (fibrosis and sclerosis) of the heart's conduction system. Sinus Sick Syndrome encompasses a range of SA node abnormalities. Our patient's likely diagnosis is due to his elderly age and variable manifestations on ECG: junctional bradycardia, asystole, AV block, and atrial tachycardia syndrome [3]. Additionally, this patient was taking Propanolol, which is also known to cause bradycardia. Patients with SA node dysfunction may be asymptomatic or present with light-headedness, dizziness, confusion, and syncope due to a transient decrease in cardiac output. Tonic or clonic seizures may also occur if there is a prolonged period of cerebral hypoperfusion [4]. However, this is a rare occurrence, and only a few cases have been reported. 

In this case, a 91-year-old man with no history of seizure disorder or epilepsy presented with classic symptoms of SA node dysfunction and had a seizure with loss of consciousness. ECG monitoring revealed apparent sinus node dysfunction. Ictal bradycardia was ruled out due to the persistence of SA node dysfunction following the syncopal events and seizures [1,5]. Propranolol was held, and a pacemaker was implanted due to symptomatic bradycardia, leading to complete resolution of his syncopal episodes and seizures. Hypoperfusion likely triggered the seizures due to severe bradycardia or sinus pause. Post permanent pacemaker, ECG monitoring showed a paroxysmal heart rate which appeared to be atrial tachycardia on the background of atrial ectopy. This suggests bradycardia tachycardia syndrome, which further elutes to SA node dysfunction and the diagnosis of Sick Sinus Syndrome. 

This case highlights the importance of accurately diagnosing a seizure episode and avoiding the unnecessary use of anticonvulsant drugs, which are pro-arrhythmic and carry a great risk of sudden cardiac death in high-risk patients. Additionally, it is important to evaluate cardiogenic causes of seizures, especially in elderly patients and those without a history of seizure disorders. The patient was discharged to follow up with neurology as an outpatient for a repeat MRI. Unfortunately, the patient was lost to follow up, and the images could thus not be obtained.

## Conclusions

Cardiac syncope and seizures are the main causes of transient loss of consciousness. At times the diagnosis can be challenging. Accurate diagnosis is crucial for successful and timely treatment and avoiding unnecessary pharmacological interventions such as anticonvulsants with broad-spectrum side effects. We urge more researchers to publish more case reports and even meta-analyses highlighting the prevalence of the disorder. 
